# Exploring the morphological dynamics of Nile tilapia (*Oreochromis niloticus* Linn. 1758) in Victoria Nile as depicted from geometric morphometrics

**DOI:** 10.1186/s40850-023-00190-9

**Published:** 2023-11-23

**Authors:** Papius Dias Tibihika, Manuel Curto, Harald Meimberg, Cassius Aruho, George Muganga, Jerome Sebadduka Lugumira, Victoria Tibenda Namulawa, Margaret Aanyu, Richard Ddungu, Constantine Chobet Ondhoro, Tom Okurut

**Affiliations:** 1 National Agricultural Research Organization (NARO), National Fisheries Resources Research Institute (NaFIRRI), Aquaculture Research and Development Centre Kajjansi (ARDC), P.O. Box 530, Kampala, Uganda; 2 University of Natural Resources and Life Sciences Vienna (BOKU), Department of Integrative Biology Research, Institute for Integrative Nature Conservation Research, Gregor Mendel Straße 33, 1180 Wien, Austria; 3 CIBIO—Research Center in Biodiversity and Genetic Resources, University of Porto, 4485-661 Vairão, Portugal, University of Porto, Porto, Portugal; 4 National Environment Management Authority (NEMA), P.O. Box 22255, Jinja Road, Kampala, Uganda; 5 Conservation, Albertine Rift Conservation Society, Kampala, Uganda; 6 Buginyanya Zonal Agricultural Research and Development Institute, NARO, P.O. Box 1356, Mbale, Uganda

**Keywords:** Nile tilapia, Geometric morphometrics, Victoria Nile, Uganda

## Abstract

**Background:**

Various anthropogenic activities continue to threaten the fish biodiversity of the East African water bodies such as the Victoria Nile. Although the Victoria Nile is a significant source of livelihood for human populations, the biology and ecology of Nile tilapia in this ecosystem remain understudied with little or no information on the morphology of the fish given varying and immense anthropogenic activities. Here, we use geometric morphometrics to examine the morphology/shape variations of Nile tilapia populations in Victoria Nile to gain insights into their current ecological state.

**Results:**

Our results indicate unexpectedly smaller Nile tilapia body weights in Victoria Nile than in *L. Victoria*. Despite this, nearly all the populations displayed a relative condition factor (Kn) of greater ≥1 suggesting a healthy stock. However, two populations, LMF and VN_Bukeeka demonstrated Kn values of less than one (< 1). We also report that some Upper and Lower Victoria Nile populations display morphological similarities. Apart from L. Albert, Nile tilapia populations from Lakes Victoria and Kyoga are morphologically divergent from the riverine ones. We note that Nile tilapia from Nalubale Dam Reservoir is morphologically distinct from the close neighbouring Victoria Nile populations which are likely allied to the influence of the Nalubale Hydroelectric power dam as a barrier.

**Conclusion:**

Nile tilapia’s morphological variation appears to be influenced by various anthropogenic disturbances notably, overfishing, hydroelectric power dams, and fish translocational history in Uganda. Management should enforce regulatory frameworks to avert human-mediated activities as these are likely to compromise the sustainability of the fisheries. Further studies are required to follow these populations with molecular genetics and environmental data to gain a deeper understanding of the fish species for informed sustainable management and conservation options.

**Supplementary Information:**

The online version contains supplementary material available at 10.1186/s40850-023-00190-9.

## Background

Like many global inland freshwater bodies, the lakes and rivers of Uganda are vital for economic development but have at the same time experienced alarming disturbances for virtually a century, since the 1920s, [1–6]. The distressing challenges have been mainly mediated by anthropogenic activities as a result of exponential demographic growth that has triggered user conflicts and escalated demands for the fisheries’ resources [7]. For instance, the high demand for fishery products led to overfishing and with a subsequent decline in fish stocks, which influenced the translocation of alien species (Nile tilapia and Nile perch) to various water bodies in Uganda [2, 8]. Other than fish translocations, Uganda’s major water bodies have been invaded by aquatic weeds which have devastated the water quality resulting in negative effects on the fisheries [9]. For instance, the water hyacinth (*Eichornia crassipes*), which was introduced in the Lakes Kyoga and Victoria as well as Victoria Nile in 1988 [6, 9, 10], and more recently (2013), the Kariba weed (*Salvinia molesta*) [11, 12] that have caused many challenges in these waterbodies. These weeds have proliferated in the water resources as a consequence of the massive influx of nutrients (mainly phosphates), propelled by various human-mediated activities [11, 13]. The anthropogenic activities have dramatically upset fish biodiversity and ecology in nearly all the country’s water bodies either directly or indirectly.

Nile tilapia in Uganda is a popular species in both aquaculture as well as capture fisheries and is thus vital for the country’s economic development. The fish is the second most economically important species after Nile perch, but the most highly valuable under aquaculture (Balirwa et al. 2003). Traditionally, Nile tilapia is non-native to Lakes Victoria, Kyoga, Nabugabo, the upper Victoria Nile, and various satellite water bodies in Uganda [2, 7, 14–16]. The species is native to Lakes Albert, Edward, George, Kazinga Channel, and the lower Victoria Nile (Uganda), and was introduced into Lakes Victoria, Kyoga, Nabugabo, and the upper Victoria Nile in the 1950s to augment the devastated capture fisheries [6, 8, 14, 17]. Albeit the introduction of Nile tilapia triggered increased fish catches, it also coincided with the many negative impacts on these water bodies [6]. These include the massive stock decline and, in some cases, extinction of the native tilapiine species such as *Oreochromis esculentus* (Singinda tilapia, Ngege) and *Oreochromis variabilis* [2, 7, 14–16]. These events coincided with the dramatic upsurge of the introduced Nile tilapia catches in the 1980s (Balirwa et al. 2003). As a result, the *L. Victoria* basin is now predominantly inhabited by three fish species notably, the native small cyprinid silverfish (*Rastrineobola argentea*) and the two non-native (introduced) species; Nile tilapia and Nile perch.

In Uganda, Lakes Victoria, Kyoga, Albert, George, and Edward are the major fish sources and generally the central focus of scientific investigations [6, 8, 18–22]. The rather less studied Victoria Nile is also a salient fish source providing employment opportunities and livelihoods to the riparian communities [23]. The Victoria Nile river drains water from *L. Victoria* to L. Kyoga and then through L. Albert to form the Albert Nile [10]. The prevalent anthropogenic activities in Vitoria Nile might have compromised the genetic integrity of the fish populations. For instance, in the 1950s, the first hydroelectric power dam, previously referred to as Owen Falls Dam and now Nalubale Dam was constructed at the source of the Nile, Jinja-Uganda [10, 24]. This dam is likely to have inhibited the geneflow of fish species between *L. Victoria* and other sections of Victoria Nile as well as L. Kyoga [10, 25]. Later (in the year 2003), another dam, Kiira, was constructed adjacent to Nalubale and perhaps exacerbated the segregation of the fish populations. In recent years, since 2007, several other hydroelectric power dams, including Bujagali and Karuma, have been constructed across the Victoria Nile, which in the long run, might pose further threats to fish biodiversity through impoundment [26, 27]. Usually, the construction of hydroelectric power dams entails the structural reservation for fish-pass-ways as well as mimicking natural bays for fish spawning [27]. It is not clear if these mitigation strategies were considered during the hydroelectric power dam construction in Uganda. Ecologically, power dams might promote fish population intraspecific divergent by blocking fish interaction or, altering habitat conditions and existing niches. These may subsequently induce selection pressures that promote changes in the organism’s behaviour and morphological variations [28].

Recently, it was observed that the genetic diversity of Nile tilapia in some section of the Victoria Nile was lower than that of other habitats such as Lake Victoria [25]. This was likely attributed to the consequence of the constructed hydroelectric power dams, loss of diversity through overfishing or water pollution, and founder effects, consistent with the historical fish translocations [25]. Nevertheless, in the study, the small sample size/scope is likely to have limited the clear understanding of the state of Nile tilapia populations given that only one site in the Victoria Nile was sampled [22, 25]. Therefore, in the current study, we sampled multiple sites in the Victoria Nile and stretched to adjacent main water bodies (Lakes). The major focus of this study was to investigate the extent of Nile tilapia morphological variation as a potential consequence of anthropogenic activities in the Victoria Nile. We approached this study by delineating the fish size and the relative condition factor variations, and compared the morphological differences amongst fish from Lakes Victoria, Kyoga, and upper and lower Victoria Nile. Further, we singled out non-native and native fish strains and subsequently tested the underlying morphological differences between the populations. The current study is intended to unveil key information vital for management and sustainable conservation decisions regarding Nile tilapia stocks in Victoria Nile River.

## Results

### Fish size and relative condition factor

**Fig. 1:**
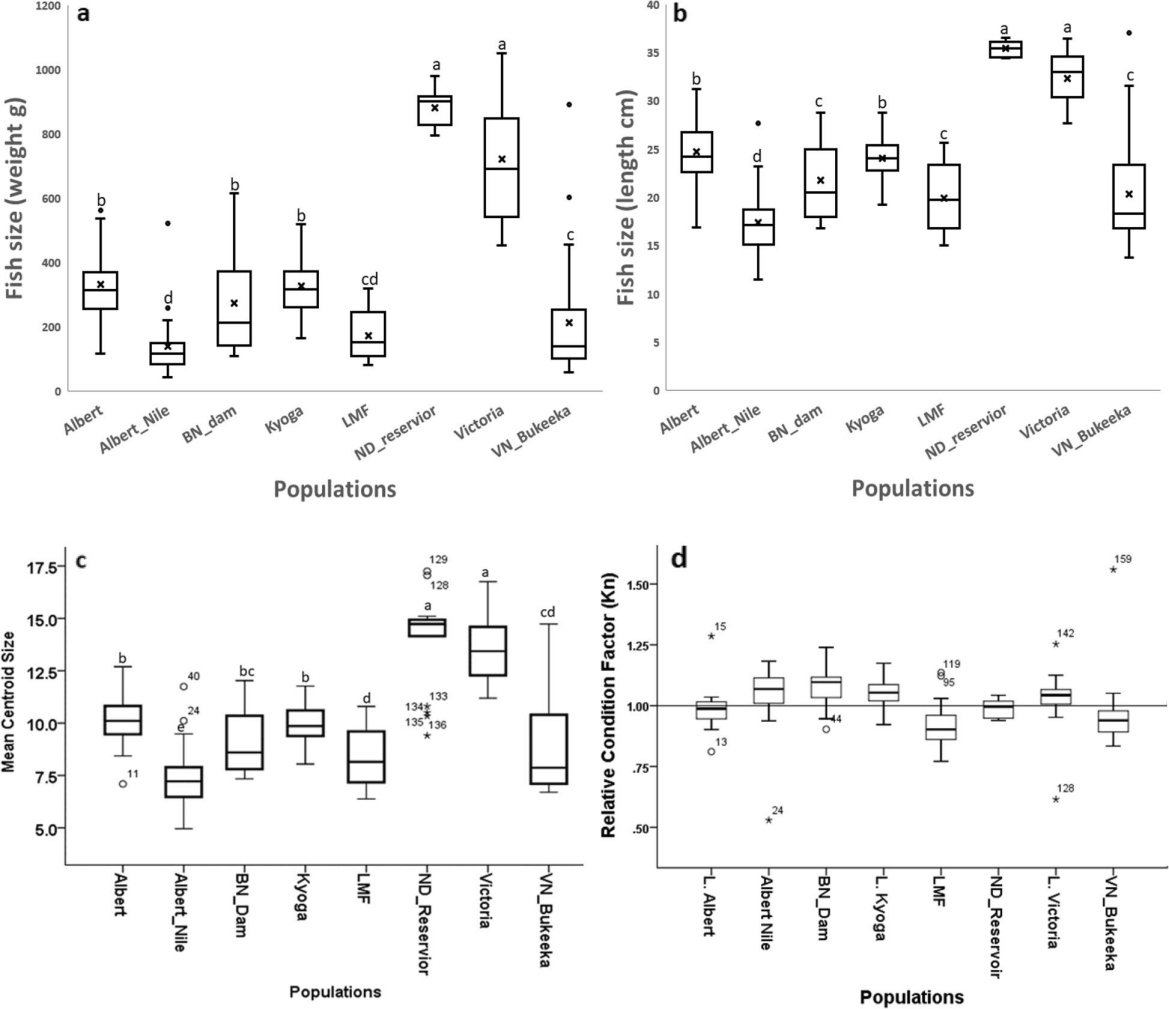
Body size variations of Nile tilapia populations, depicting weight (**a**), length (**b**), Centroid Size (**c**), and condition factor (**d**). Different superscript letters indicate statistically significant different values (*p* < 0.05) and vice versa

The comparisons of Nile tilapia populations based on body sizes; weight (g), length (cm), and the centroid size (CS), from the 8 populations indicated that ND_Reservoir and *L. Victoria* individuals had relatively larger body weights and were significantly differentiated from the remaining populations (*p* < 0.05). No significant divergence was found among the remaining populations, with LMF and Albert_Nile showing the smallest sizes (Fig. 1). Generally, ND_Reservoir, and Albert_Nile indicated the least within population body size variance, mainly considering weight (g) (Fig. 1a). Similarly, considering the fish size (length and CS), Only the ND_Reservoir demonstrated the least size variance within population (Fig. 1b; c). Apart from ND_Reservoir and *L. Victoria*, the Victoria Nile populations unveiled the smallest body weights and CS of less than 400 g and 10 respectively (Fig. 1). L. Albert,Albert_Nile, BN_Dam, L. Kyoga, ND_Reservoir, and *L. Victoria*, generally revealed Kn values equal or greater than one (Kn ≥ 1). The remaining two populations: LMF and VN_Bukeeka indicated Kn values of less than one (Kn < 1) (Fig. 1d).

### Geometric morphometrics

**Fig. 2:**
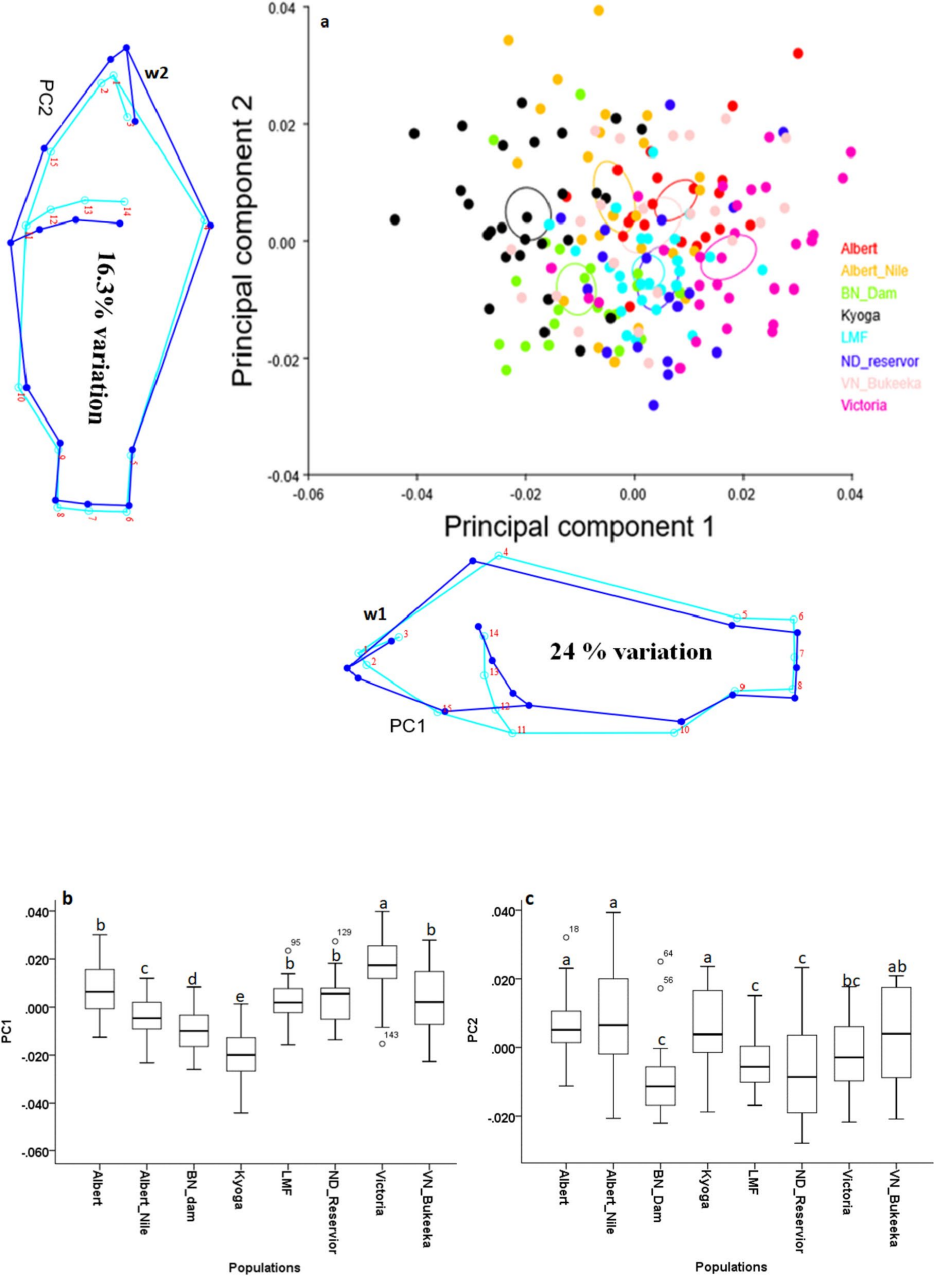
Illustration of PCA for the 8 Nile tilapia populations depicted from principal component (PC) scores. a represents the PCA scatter plot, w1, and w2 depict wireframes for PC1 and PC2 respectively, demonstrating deformations for the major features behind the morph variations. Wireframe colours; light green and light blue, show the ideal shape and shape change respectively. Ellipses in the scatter plot represent a 95% confidence interval for the means. b and c depict PC1 and PC2 scores plotted on bar graphs respectively. Different superscript letters on the bar graphs indicate statistically significant Nile tilapia shapes (*p* < 0.05) and vice-versa

PC1 and PC2 accounted for 24% and 16.3% of the shape variation respectively (Fig. 2). Nile tilapia population from *L. Victoria* displayed higher and significant shape divergent (*p* < 0.05), followed by VN_Bukeeka, ND_Reservoir, LMF, and L. Albert, with L. Kyoga indicating the least feature changes as represented on PC1 (Fig. 2, b). On PC2, L. Albert, Albert Nile, L. Kyoga, and VN_Bukeeka presented pronounced shape feature divergence compared to the other populations. On PC2, *L. Victoria* indicated shape feature variability analogous to ND_Reservoir, LMF, and BN_Dam (Fig. 2 a; c). Shape changes on PC1 were mainly associated with the head-to-body depth and the caudal peduncle, without shape deviations on the caudal fin origin, describing a downward pointing head and narrowing body depth, (Fig. 2 PC1; w1), see also the supplementary materials, Fig. S.1. On the other hand, PC2 indicated body feature changes linked with mainly the head and body depth, with relatively shape deformations on the caudal fin origin, describing an upward-pointing head and body widening (Fig. 2 PC1; w2).

**Fig. 3:**
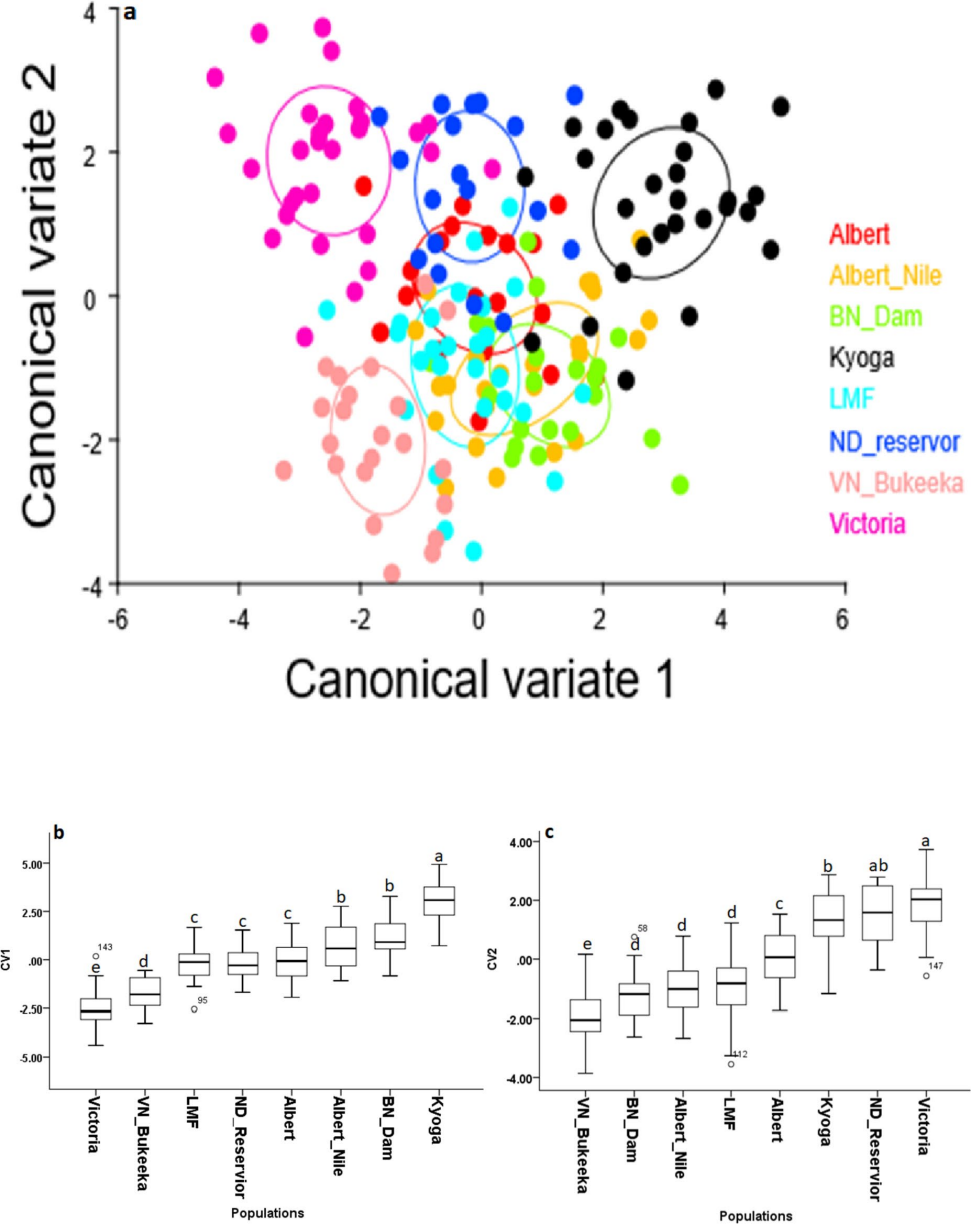
Illustration of CVA for the 8 Nile tilapia populations, depicted from canonical variate (CV) scores. a represents CVA scatter plot, while b and c portray CV1 and CV2 scores plotted on bar graphs respectively. Ellipses in the scatter plot represent a 95% confidence interval for the means set at 10,000 iterations. Similar superscript letters on the bar graphs indicate that populations are morphologically clustered together and therefore undifferentiated significantly (*p* < 0.05) and vice-versa

**Fig. 4:**
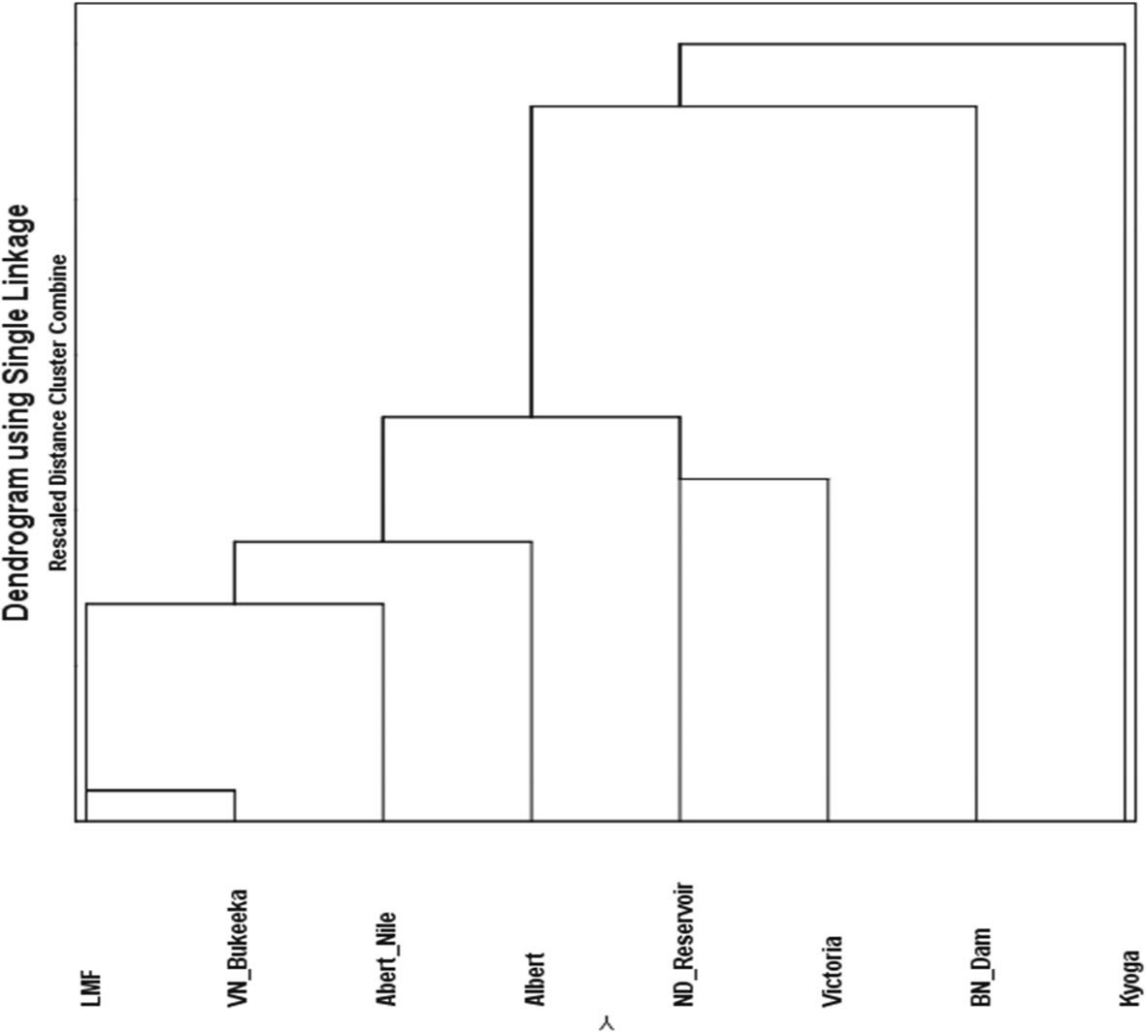
Dendrogram illustrating the Nile tilapia population clusters derived from the Procrustes coordinates

Based on CV1, Lakes Kyoga and Victoria as well as VN_Bukeeka were morphologically divergent, despite relatively overlapping (Fig. 3, a). On the same axis, two morphotype clusters i) LMF, ND_Reservior, and L. Albert, and ii) BN_Dam and Albert Nile, were observed (Fig. 3 a; b). However, concerning CV2, more clusters were revealed including, i) LMF and Albert Nile and ii) L. Kyoga and ND_Reservior, iii) *L. Victoria* and ND_Reservior, iv) Albert Nile and L. Albert, v) VN_Bukeeka and Albert Nile, and vi) VN_Bukeeka and LMF). On this axis (CV2), the other populations were indicated as morphologically distant (Fig. 3). Despite some overlap, indicated by CV2, generally, BN_Dam and L. Kyoga, were morphologically distinct (Fig. 4). Generally, results regarding population shape differentiation based on CVA were congruent with the dendrogram (Fig. 4).

**Fig. 5:**
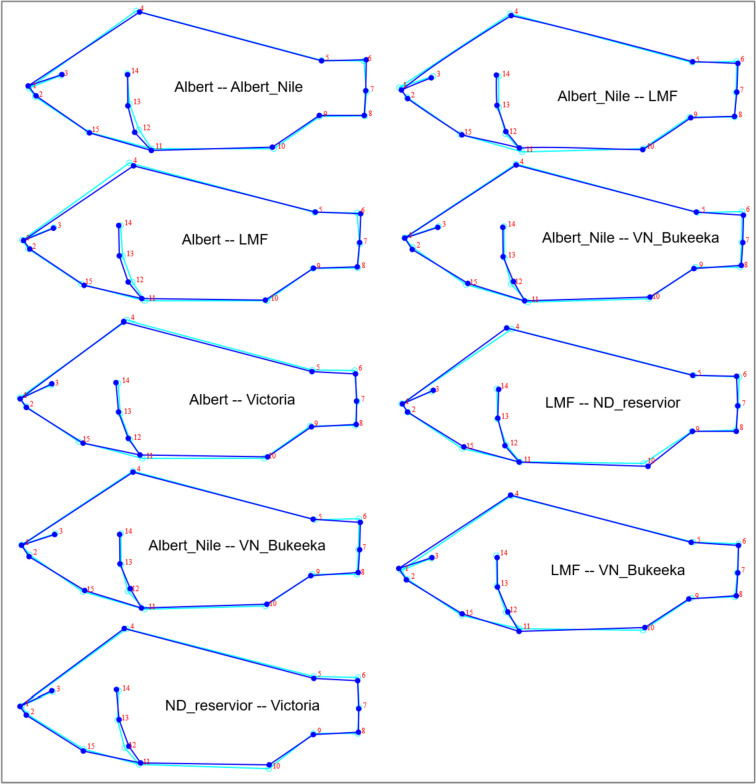
Population pairwise comparison of Nile tilapia morphotypes based on discriminant function analysis (DFA). The wireframe colours; light green and light blue, represent the shapes of the compared population pairs. The uniformity of the wireframes indicates similar Nile tilapia population morphotypes

Following the population pairwise comparisons based on discriminant function analysis (DFA), the results were generally consistent with CVA and Dendrogram. Here, DFA grouped nine morphologically similar population pairs (Fig. 5) and 20 divergent population pairs (see supplementary materials Fig. S.2). Interestingly, like, in CVA and dendrogram, the individual shapes in L. Kyoga and BN_Dam population morphotypes were consistently distanced from the other populations. L. Albert Nile tilapia morphotypes showed close similarity with all the lower Victoria Nile (L. Albert, and LMF) as well as with those of the upper Victoria Nile (VN_Bukeeka and ND_Reservoir, including *L. Victoria*), apart from BN_Dam and L. Kyoga (Fig. 5). The upper Murchison Falls Victoria Nile populations (VN_Bukeeka, BN_Dam, and ND_Reservoir) did not appear to associate with each other.

**Fig. 6:**
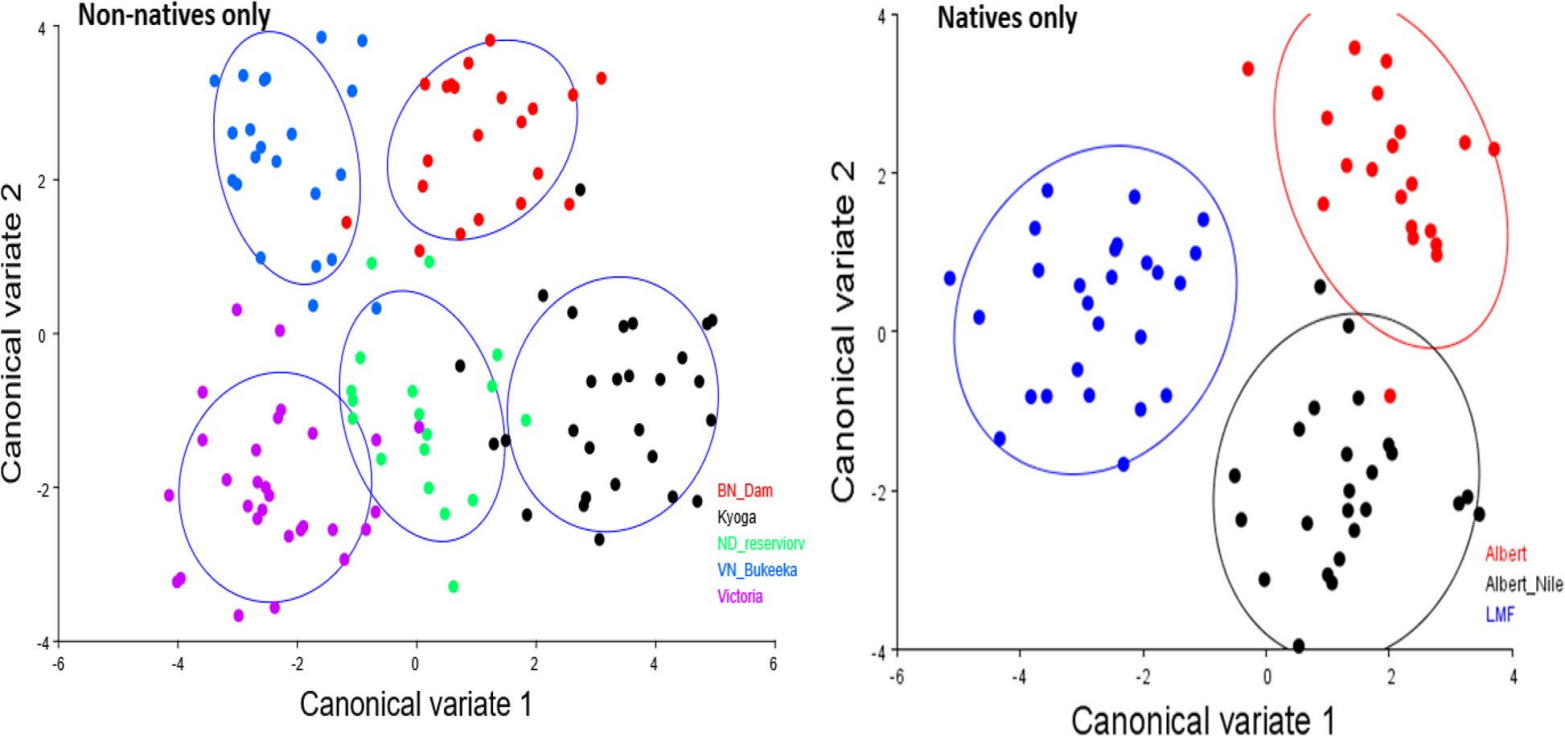
CVA results comparing the morphology between nonnative and native Nile tilapia populations

To further gain insights into the extent of morphological divergence between the different categories of Nile tilapia populations, we separated the groups into two; native and non-native, and subjected them to CVA within groups. Results showed somewhat clear morphotype structure (Fig. 6). Albeit the non-native Nile tilapia populations were indicated relatively overlapping, the native CVA demonstrated clearly separate morphotype groups (Fig. 6).

## Discussion

### Fish size and Kn

Our findings show significant differences between Nile tilapia populations based on the body size and the Kn in the studied Victoria Nile ecosystem. We observed a larger body size of Nile tilapia in *L. Victoria* and ND_Reservoir compared to lower body sizes in the other populations. This was interesting, particularly, given that ND_Reservoir neighbours BN_Dam, suggesting the likely impact of the Nalubale dam on the ecology of Nile tilapia in the ecosystem. In general, all the sampled sites in Victoria Nile demonstrated small body sizes. Albeit one may reckon that the sampling artifacts may contribute to the observed variations, centroid size generally demonstrated consistent values.

The observed size variations might be linked with some anthropogenic impacts on Victoria Nile, particularly overfishing, and habitat degradation albeit other factors such as hydroelectric power dams, and water flow rate may play pivotal roles [29]. It is widely known that usually fishing targets large-bodied species and as the fishing effort intensifies, the fish size at maturity reduces which may elucidate the current data [29–31]. Overfishing or high fishing mortality exacerbates the contemporary evolution towards the increased allocation of earlier energy to reproduction and consequently smaller-sized fish at age [31]. These findings may be correlated with the previous reports which indicate that overfishing is the major cause of the dwindled fish stocks and subsequent fishery collapse in the Lake Victoria basin [15, 17]. It should thus be noted that the relatively large size Nile tilapia in *L. Victoria* compared to the riverine populations might suggest that overfishing may be more detrimental in the riverine environs than in wide and open water bodies. For instance, while fisheries activities in large water bodies for example *L. Victoria*, can be regulated by restricting fishing in certain areas (e.g., inshore breeding areas) to allow fish stocks to replenish, it can be problematic and confounding in smaller-sized water bodies like the case of Victoria Nile. This is because riverine fishing activities cannot be localized (e.g., only in the deeper/offshore waters), rather fish harvest is usually conducted throughout the water body, which does not spare areas, important for breeding.

However, one salient point to note is that overfishing in Victoria Nile might have also been influenced by the existence of hydroelectric power dams (HEP). Fundamentally HEP across water bodies serve as fish barriers that subsequently confine the organisms in limited habitats. In Victoria Nile, the HEP might have confined Nile tilapia populations to limited refugia and hence exposing the fish’s vulnerability to easy and high fishing mortality (overexploitation). Albeit there are also reports of overfishing in *L. Victoria* [6, 15, 32], the vast openness of water to fish movement coupled with restricted fishing areas (inshore waters) may provide limited chances for overexploitation. Overall, these results are congruent with the previous studies on Nile tilapia in which indicators of genetic bottleneck were observed in some section of the Victoria Nile river (despite one sampled site), suspected to overfishing [25]. It should be noted that recently more HEP facilities have been constructed along the Victoria Nile stretch and these are likely to impose more unprecedented threats to fish biodiversity [33].

Despite the significant variation in the Kn as noted amongst the studied Nile tilapia populations, the overall results demonstrated healthy fish stocks, part from a few stocks. The Kn results are consistent with those of [22, 34] who correlated this to the rich trophic state of the water body. The fact that the majority Nile tilapia populations indicated relatively commendable Kn results, these might elucidate that the Nile tilapia populations grow isometrically [35]. However, the low Kn values observed in LMF and VN_Bukeeka may be a consequence of anthropogenic activities including water pollution and habitat loss, among others [36].

### Geometric morphometrics

Divergent and significant morphotype changes were generally observed in the lakes; *L. Victoria* based on PC1, as well as Kyoga, Albert, and Albert Nile on PC2, compared to the other populations in the riverine environment; Victoria Nile populations. Importantly, the current study also showed that generally apart from BN_Bukeeka and L. Kyoga, the Nile tilapia morphotypes from the upper Nile (including *L. Victoria*) were morphologically similar to the lower Nile populations. It is expected that shape feature changes in the lotic conditions might generally differ from the lentic environment due to the continuous stressing conditions of the fast water current [37]. Additionally, the prevailing natural fish barriers like the Murchison Falls radically would play a pivotal role to detach the upper Nile populations from the lower ones [38], thus defining different morphotypes. These scenarios appear to have no significant role in the morphology of the current studied populations.

This study potentially informs that the observed morphotype clusters might be influenced by the historical anthropogenic fish translocations between the various water bodies in Uganda [5, 6, 8, 38]. Fish translocation in Uganda began in the 1950s through which several tilapiine species, including Nile tilapia, were relocated into Lakes Victoria and Kyoga basins from L. Albert [14, 38, 39]. These reports were supported by both molecular genetics and geometric morphometric studies [22, 25, 39]. It is thus likely that the closely related morphotypes between the L. Albert basin (Lower Victoria Nile) with those of the upper Nile might be a consequence of shared genotypes following the historical translocations. However, the observed distant morphotype of L. Kyoga might be associated with the genetic founder effects as detected in the previous studies [25] following the past restocking programs. Similarly, the distinct Below Nalubale Dam morphotype might be a result of geneflow barrier caused by the hydroelectric power dams coupled with divergent abiotic conditions and anthropogenic disturbances. Although some of these populations have been analysed genetically, a study encompassing all of them is still lacking. The future comprehensive genetic investigations on Nile tilapia in the Victoria Nile will contribute important insights about the the structure of Nile tilapia stocks in the waterbody.

Nile tilapia shape variations were mainly associated with the head-to-body depth and the caudal peduncle, describing a downward-pointing head and narrowing body depth. These observations might be indicators of re-adjusting the body forms for suitability and survival in varying environments. Principally, the shape orientation, size, and structure of the body parts may permit different or the same fish species to live in varying habitats or different locations in the same environment. Thus, the phenotypes or external anatomy of a fish may unveil a great deal about how and where it lives. For instance, responses to predator-prey avoidance (e.g., pressure from Nile perch), and fast-flow water currents, inter alia, might contribute to morphological variations [37, 40, 41]. Environmental parameters such as temperature, oxygen, depth, water current/flow rates, and eutrophication levels were not measured during this work. These should be considered in future studies since they may explain some of the observed shape variations.

More importantly, the morphotype divergence of ND_Reservoir from the neighbouring upper Victoria Nile populations, particularly BN_Dam, maybe a clear indicator of population detachment through the establishment of Nalubale HEP. Apart from the observed morphological differentiation, the Nalubale HEP might have also altered the genetic integrity of the populations which can subsequently be detrimental to the populations. This is consistent with the observed small-sized Nile tilapia in Victoria Nile and the previous studies in which the indicators of genetic bottleneck and genetic drift coupled with low genetic diversity were encountered in the river [25].

Results from the current study also showed that the native Nile tilapia populations were more morphologically divergent among each other than non-native ones. The less morphologically divergent Nile tilapia populations particularly observed in the non-native populations may suggest similar Nile tilapia strains promoted by the historical fish translocations in Uganda [5, 6, 25, 42].

In principle, the Victoria Nile fisheries particularly the upper Murchison Falls, are less or insufficiently studied [36, 43]. Therefore, it is apparent that the fish stocks and perhaps the water quality of this important aquatic system have been compromised which might require crucial management strategies for the sustainability of the ichthyofauna therein.

## Conclusion and recommendations

Apart from a few populations, generally the Nile tilapia stocks in Victoria Nile appear in a healthy state based on the Kn results. We detected relatively smaller fish sizes that may be attributed to various anthropogenic activities which are subject to further exploration. The Nile tilapia morphological similarity observed between the upper and lower Victoria Nile may be related to same stocks propelled by the historical fish translocations in Uganda. Importantly, we noted a significant variation of both body size and morphology between ND_Reservoir and the close neighbours in the Victoria Nile (below Nalubale Dam) which may be a consequence of the barrier, Nalubale Dam. With time, the additional newly constructed dams across the Victoria Nile might compromise the genetic integrity of the Nile tilapia stock thereby threatening the sustainability of the species in the ecosystem. While several factors may be responsible for the observed findings in the current study, we recommend future studies to holistically focus on the environmental parameters and anthropogenic activities, combined with molecular data to explore the Victoria Nile ecosystem. This will provide salient notions to inform management and conservation options of the fish stocks in Victoria Nile.

## Materials and methods

### Study area

**Fig. 7:**
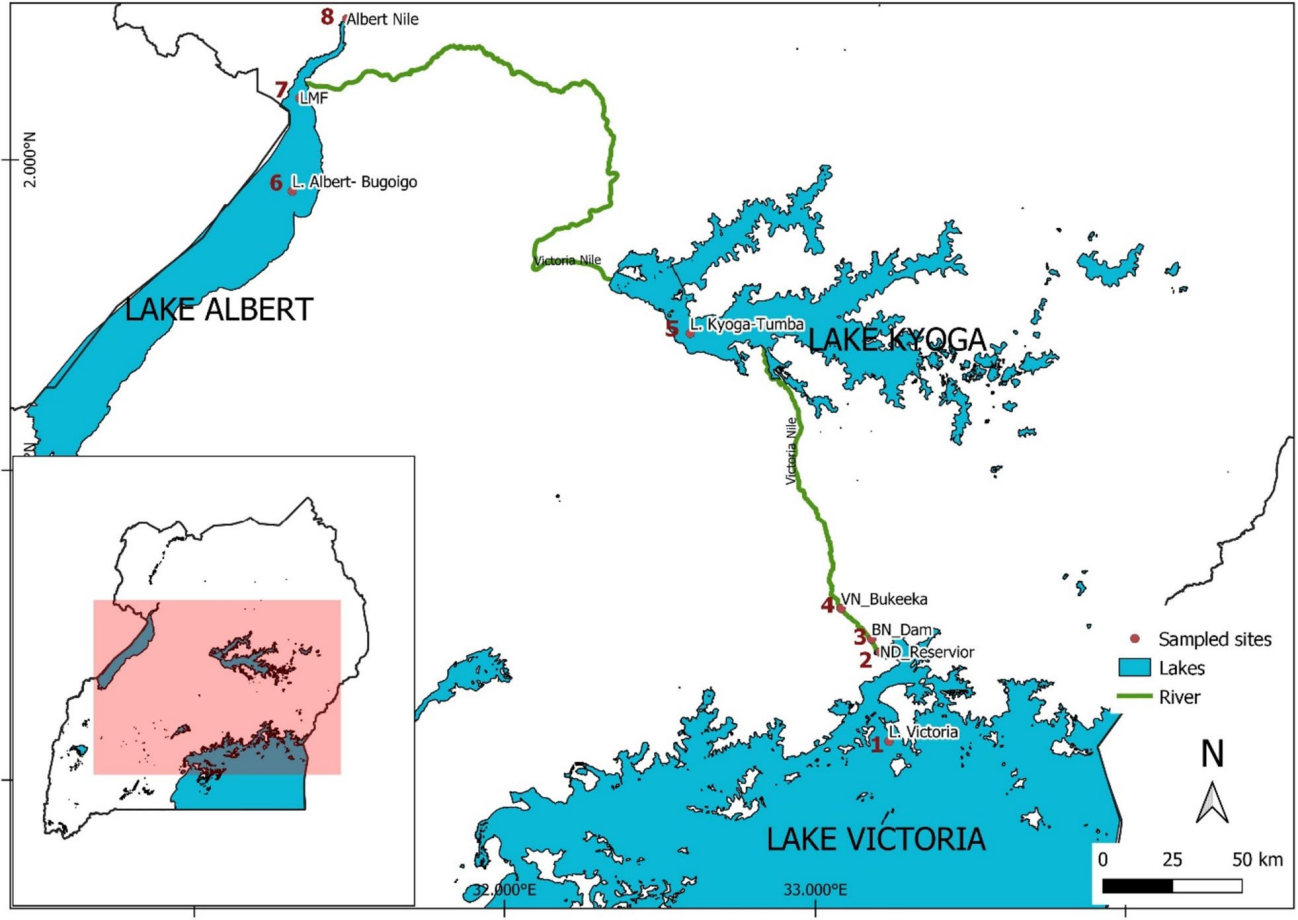
Collection sites of Nile tilapia samples. Numbers from 1 to 8 indicate sample collection sites. The Nalubale hydroelectric power dam lies between sites 2 and 3. See also Table 1 for specific site descriptions

Nile tilapia samples were collected mainly from Victoria Nile, with additional specimens from Lakes Victoria, Kyoga, and Albert as well as the Albert Nile (Fig. 7). Field excursions were conducted between January and February 2020.

### Field sampling

At the time of field data collection, the Government of Uganda had temporarily designated a quota fishing season for the Victoria Nile system to allow fish recovery following overfishing threats. In this case, before sampling, verbal fishing permission was acquired from the Uganda People’s Defence Forces (UPDF), who were guarding the water bodies in Uganda at the time. Fish samples were collected using experimental gillnets (127 mm mesh size), set during the day. Fishing was carried out with due diligence by avoiding any artifacts that could lead to fish shape malformations. Since fish landed when freshly dead, no special animal rights were observed/required. On landing, fresh fish specimens were quickly weighed (g) using a digital weighing scale, measured for total length (cm) using a ruler, and subsequently photographed with a digital camera (Canon IXUS 275 HS, 12x optical zoom). The capture of digital images followed the guidelines from Tibihika, Waidbacher [22]. A total of 196 individuals from 8 populations were sampled and analyzed (Fig. 7; Table 1). It should be noted that the Nalubale dam reservoir and the below Nalubale dam sampling sites were once one waterbody without any interruptions but are now separated by the Nalubale dam hydroelectric power plant (see Fig. 7; between sites 2 and 3).

**Table 1:** Details of sample sources and sizes

S/No	Sample source/identity	District/site	Sample size	Coordinates	
1	L. Victoria	Jinja	30	0.124216	33.237244
2	ND_Reservior	Jinja	17	0.412424	33.208296
3	BN_dam	Jinja	21	0.453489	33.181493
4	VN_Bukeeka	Kayunga	29	0.552432	33.082585
5	L. Kyoga	Nakasongola-Tumba	30	1.440512	32.595943
6	L. Albert	Buliisa-Bugoigo	20	1.898907	31.315673
7	LMF	Buliisa-Wanseko	26	2.199049	31.340102
8	Albert_Nile	Pakwach-Kalolo	23	2.45354	31.491157

*S/No* Serial number, *ND* Nalubale Dam, *BN* Below Nalubale, *VN* Victoria Nile, *LMF* Lower Murchison Falls

## Analyses

### Fish relative condition factor (Kn)

The relative condition factor/coefficient of condition (Kn) may be regarded as the general measure of the physical health of fish based on the assumption that heavier organisms of a given length are in better condition [44]. Kn also commonly referred to as Fulton’s condition factor, is considered a useful approach for depicting the organisms’ (fish) physiological status and may relatively be employed as a tool for fisheries management [35, 44, 45]. Since Kn is directly congruent with weight, it can be pertinent and salient in assessing whether an organism is making good use of its environmental trophic resources. Kn is defined from the expression; Wo/Wc, where Wo is the observed weight and Wc is the calculated weight [45]. Good fish condition is deduced when Kn ≥1, while when Kn < 1 explains poor fish condition in the environment [46]. Therefore, in the current study, to gain insight into the broad overview of the performance of the Nile tilapia populations in Victoria Nile, we calculate Kn based on the expression; $Kn=\frac{Wo}{Wc}$.

### Geometric morphometrics: landmark digitization

**Fig. 8:**
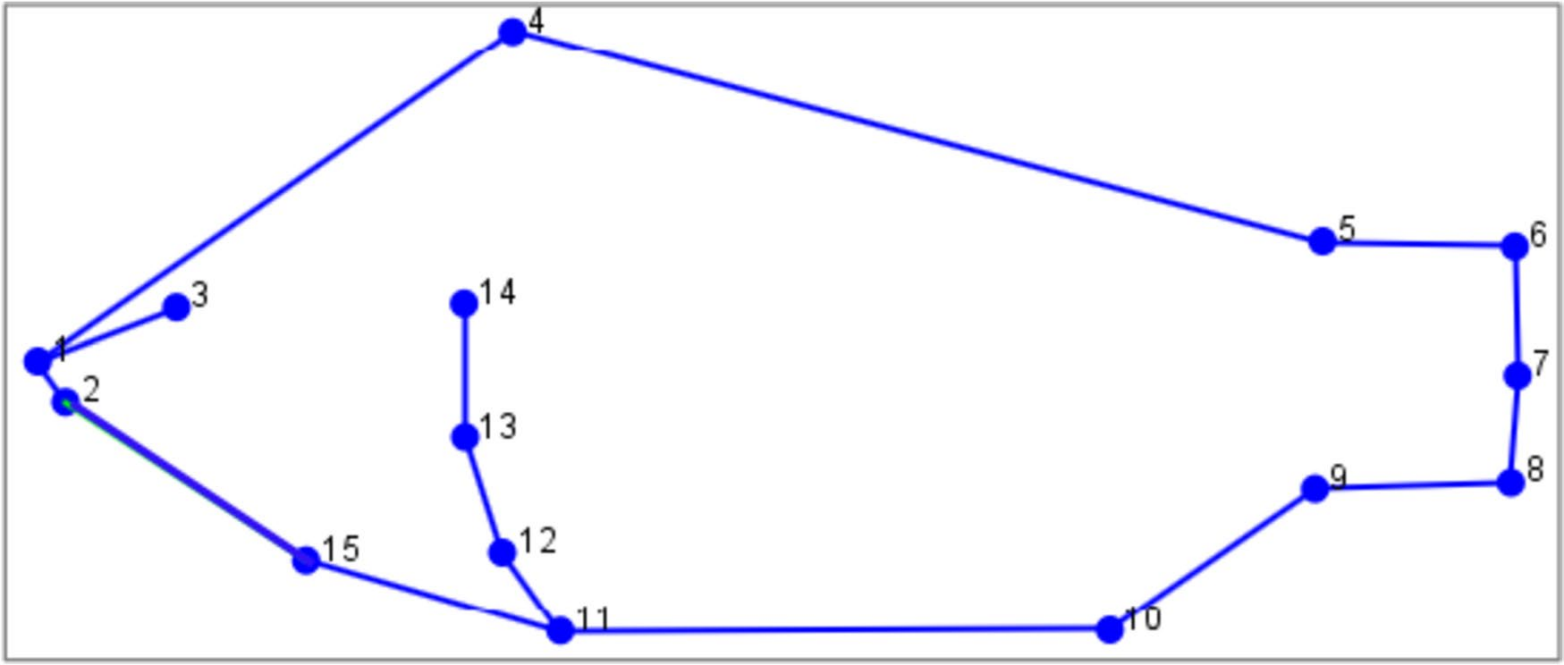
Specimen wireframe illustrating the positions of 15 homologous landmarks: (1) anterior tip of the snout with mouth closed, (2) posterior end of the mouth, (3) Orbit/eye center, (4 & 5) anterior and posterior insertions of the dorsal fin, (6) the dorsal origin of the caudal fin, (7) mid-dorsal and ventral origin of the caudal fin, (8) the ventral origin of the caudal fin, (9) posterior insertion of the anal fin, (10) the anterior origin of the anal fin, (11) the anterior origin of the pelvic fin, (12) the posterior origin of the pectoral fin, (13) the anterior origin of the pectoral fin, (14) most posterior end of the operculum, and (15) juncture of the ventral edge of the operculum [22, 50]

Digitization of landmarks followed the procedures in Tibihika, Waidbacher [22]. Landmark acquisition was conducted using two thin-plate spline programs (Tps): TpsUtil (utility) and TpsDig (digitizer) [47, 48]. TpsUtil was used to build Tps files that were imported into TpsDig for digitizing, sequentially 15 homologous landmarks (Fig. 8) to subsequently generate two-dimensional x and y coordinates [22, 48, 49]. Landmark digitization was performed by one scientist to enhance consistency and error minimization. The anatomical description of each landmark is presented in Fig. 8.

### Statistical analysis

To test the effect of a given site/locality on the size (weight and length) and condition factor of Nile tilapia population variations, we used a One-Way ANOVA implemented in SPSS IBM version 21. Here, weight and length as well as the condition factor variables were taken as explanatory variables and population sites or locations as independents.

Nile tilapia shape variations were statistically investigated using the MorphoJ program, version 1.07a [51], freely downloadable from http://www.flywings.org.uk/MorphoJ_page.htm. Here, the x and y coordinates generated by the TpsDig, were imported into MorphoJ for subsequent shape extraction based on Procrustes superimposition [22]. Procrustes analysis is vital for aligning the landmarks and for filtering any variations that may arise from different specimen sizes between the specimens [37]. Following this analysis, a covariance matrix was generated from which various multivariate analyses including, Principal Component Analysis (PCA), Canonical Variate Analysis (CVA), and Discriminant Function Analysis (DFA) were performed. PCA was conducted to investigate and display the main features responsible for shape variation based on different Nile tilapia populations. Here, the first principle component (PC1) delineates the highest amount of variation whilst the second component (PC2) defines the next highest variability, etc. until variability becomes less vital to depict data [52]. To optimize the visualization of shape feature changes based on PCA, we exported principal component scores to the SPSS program to test the effect of site/habitat on the shape variability using One-Way ANOVA procedures. CVA was carried out to portray information on the shape features that best distinguish between multiple groups of Nile tilapia through clustering. Related to PCA, we exported canonical variate scores to the SPSS program to enhance the visualization and validation of shape separation based on populations using One-Way ANOVA procedures.

To validate and enhance the effect of habitat/population site on the size variation, we analyzed centroid size (CS) variables based on MorphoJ program procedures. CS is a composite size measure based on all landmarks and is proportional to the square root of the summed squared inter-landmark distances that are employed to estimate body size in geometric morphometrics [37, 53]. CS was calculated following the procedures of Procrustes superimposition [37, 51].

To further assess the nature of morphological divergence amongst Nile tilapia populations, we performed DFA for population pairwise comparisons based on wireframes. We further validated the results arising from CVA and DFA by using the SPSS program to construct a dendrogram derived from the MorphoJ averaged Procrustes coordinates program. Because the upper Murchison Falls Victoria Nile, including Lakes Kyoga and Victoria, are radically inhabited by the introduced stocks of Nile tilapia populations contrary to the lower Nile [38], we also compare the CVA results of the Native and non-native strains. Here the native Nile tilapia populations include LMF, Lake Albert, and Albert Nile. The non-natives involve Lakes Victoria and Kyoga, Nalubale Dam Reservoir, Below Nalubale Dam, and Victoria Nile Bukeeka.

### Supplementary Information

**Additional file 1.**

## Data Availability

The datasets generated and/or analyzed during the current study are not publicly available due to the big size nature of the data that cannot be uploaded as a supplementary file. However, the dataset is available from the corresponding author on request.
