# Supplementary material for: Exploring the morphological dynamics of Nile tilapia (Oreochromis niloticus Linn. 1758) in Victoria Nile as depicted from geometric morphometrics
Source: BMC Zool. 2023 Nov 23;8:28. doi: 10.1186/s40850-023-00190-9 (PMC10668481; doi:10.1186/s40850-023-00190-9)
Supplement: Supplementary file 1 — Additional file 1. [file 40850_2023_190_MOESM1_ESM.docx]

**Exploring the morphological dynamics of Nile tilapia (*Oreochromis niloticus* Linn. 1758) in Victoria Nile as depicted from geometric morphometrics.**

Papius Dias Tibihika^1,2*^, Manuel Curto^2,4^, Harald Meimberg^2^, Cassius Aruho^1^, George Muganga^3^, Jerome Sebadduka Lugumira^3^, Victoria Tibenda Namulawa^1^, Margaret Aanyu^1^, Richard Ddungu^1^, Constantine Chobet Ondhoro^5^_,_ Tom Okurut^3^

^1^National Agricultural Research Organization (NARO), National Fisheries Resources Research Institute (NaFIRRI), Aquaculture Research and Development Centre Kajjansi (ARDC) P.O. Box 530 Kampala, Uganda. [papiust@yahoo.com](mailto:papiust@yahoo.com), [aruhoc@gmail.com](mailto:aruhoc@gmail.com), [tibendaviki@gmail.com](mailto:tibendaviki@gmail.com), [ddungurichard@yahoo.com](mailto:ddungurichard@yahoo.com)

^2^University of Natural Resources and Life Sciences Vienna (BOKU), Department of Integrative Biology Research, Institute for Integrative Nature Conservation Research, Gregor Mendel Straße 33, 1180 Wien, Austria [manuel.curto@boku.ac.at](mailto:manuel.curto@boku.ac.at), [meimberg@boku.ac.at](mailto:meimberg@boku.ac.at)

^3^National Environment Management Authority (NEMA), P.O. Box 22255 Jinja Road, Kampala-Uganda.

[george.muganga@nema.go.ug](mailto:george.muganga@nema.go.ug), [jerome.lugumira@nema.go.ug](mailto:jerome.lugumira@nema.go.ug), [tookurut@gmail.com](mailto:tookurut@gmail.com)

^4^MARE, Marine and Environmental Sciences Center, Faculty of Sciences, University of Lisbon, Lisbon, Portugal, [macurto@fc.ul.pt](mailto:macurto@fc.ul.pt)

^5^Buginyanya Zonal Agricultural Research and Development Institute, NARO, P.O. Box 1356, Mbale, Uganda.

[occonstantine88@gmail.com](mailto:occonstantine88@gmail.com)

**Supplementary materials**

**Figure S. 1.** Morphological feature changes for specific populations depicted from PC1

**
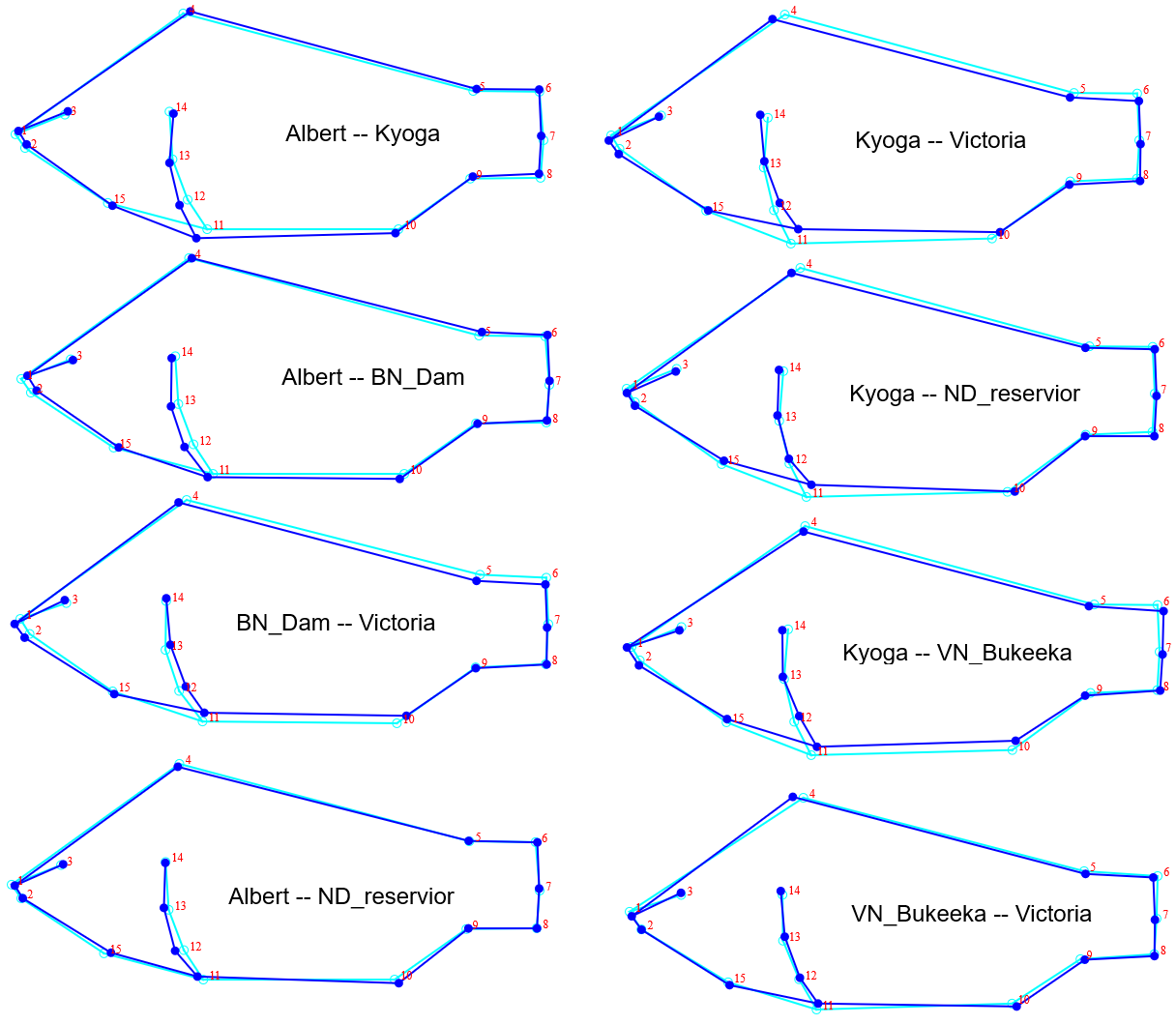
**

**
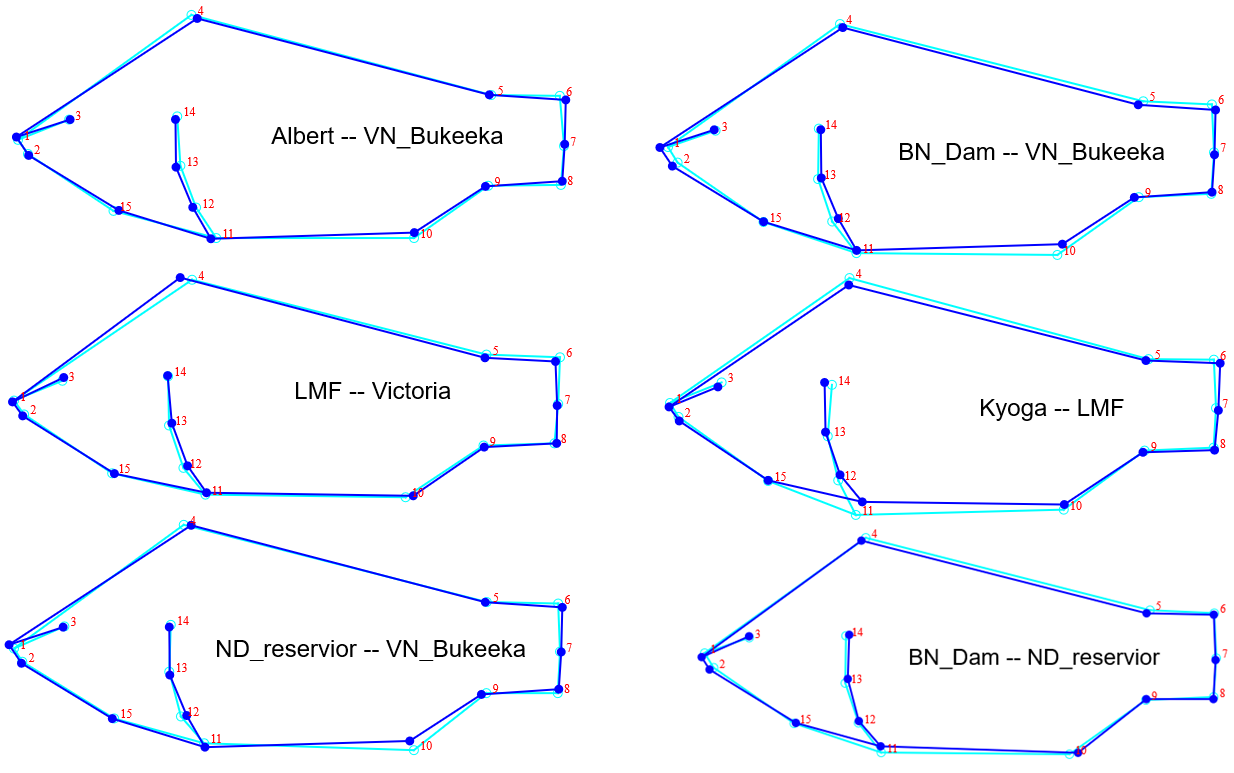
**

**
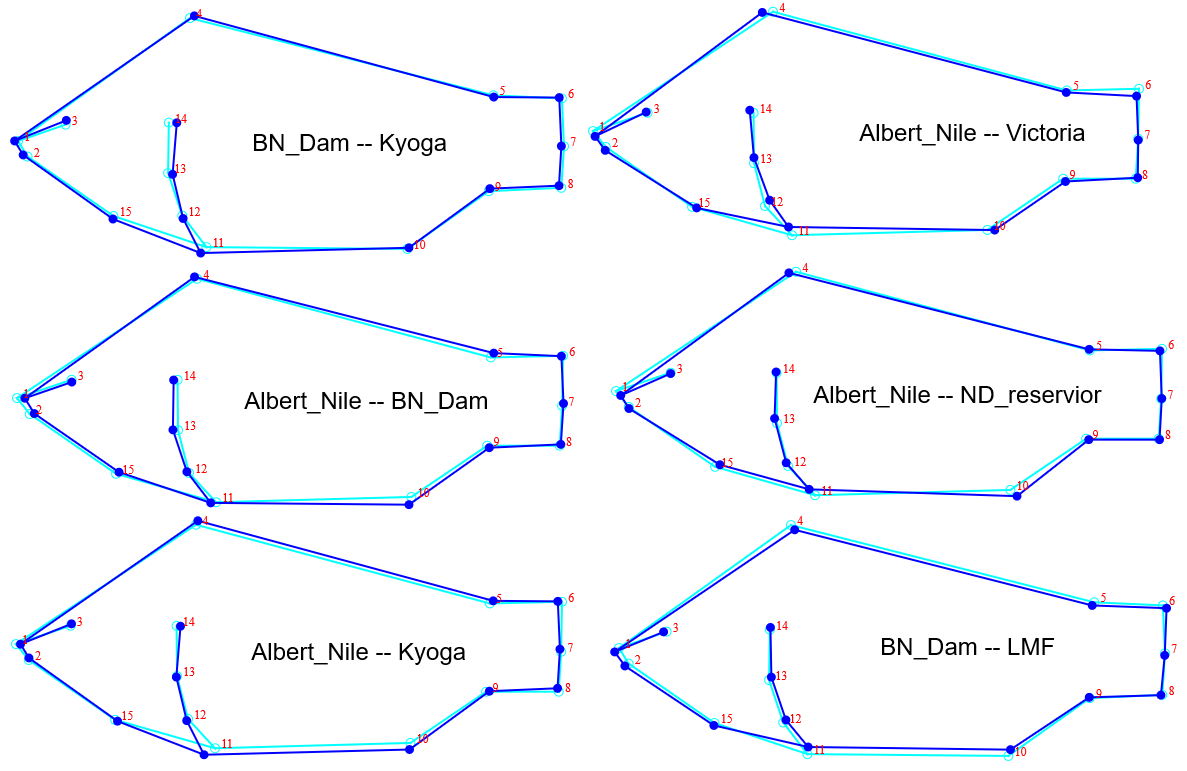
**

**Figure S. 2.** Population pairwise comparison of Nile tilapia morphotypes based on discriminant function analysis (DFA).
